# Cellular Therapy for the Treatment of Paediatric Respiratory Disease

**DOI:** 10.3390/ijms22168906

**Published:** 2021-08-18

**Authors:** Laura C. Brennan, Andrew O’Sullivan, Ronan MacLoughlin

**Affiliations:** 1 College of Medicine, Nursing & Health Sciences, National University of Ireland, H91 TK33 Galway, Ireland; L.BRENNAN22@nuigalway.ie; 2 Research and Development, Science and Emerging Technologies, Aerogen Limited, Galway Business Park, H91 HE94 Galway, Ireland; aosullivan@aerogen.com; 3 School of Pharmacy and Pharmaceutical Sciences, Trinity College, D02 PN40 Dublin, Ireland; 4 School of Pharmacy & Biomolecular Sciences, Royal College of Surgeons in Ireland, D02 YN77 Dublin, Ireland

**Keywords:** paediatric, cellular therapy, respiratory disease, aerosol delivery, inhalation, stem cells, extracellular vesicles, PARDS, asthma, interstitial lung disease

## Abstract

Respiratory disease is the leading cause of death in children under the age of 5 years old. Currently available treatments for paediatric respiratory diseases including bronchopulmonary dysplasia, asthma, cystic fibrosis and interstitial lung disease may ameliorate symptoms but do not offer a cure. Cellular therapy may offer a potential cure for these diseases, preventing disease progression into adulthood. Induced pluripotent stem cells, mesenchymal stromal cells and their secretome have shown great potential in preclinical models of lung disease, targeting the major pathological features of the disease. Current research and clinical trials are focused on the adult population. For cellular therapies to progress from preclinical studies to use in the clinic, optimal cell type dosage and delivery methods need to be established and confirmed. Direct delivery of these therapies to the lung as aerosols would allow for lower doses with a higher target efficiency whilst avoiding potential effect of systemic delivery. There is a clear need for research to progress into the clinic for the treatment of paediatric respiratory disease. Whilst research in the adult population forms a basis for the paediatric population, varying disease pathology and anatomical differences in paediatric patients means a paediatric-centric approach must be taken.

## 1. Introduction

Respiratory disease is a leading cause of mortality worldwide and is estimated to account for 4 million deaths globally each year, with children being considered extremely susceptible [1]. Over 6.6 million children under the age of 5 die annually, with respiratory disease being the leading cause of death [2]. Asthma is the most common chronic respiratory disease of childhood, currently estimated to affect 14% of children globally, with its prevalence increasing [2]. Cystic fibrosis is the second most common paediatric chronic respiratory disease, estimated to affect between 1:3000 and 1:4000 live births globally [3]. Advances in prenatal and postnatal care have led to increased survival rates and a reduction in respiratory comorbidities in preterm infants [4]. However, the consequences of lung immaturity including development of bronchopulmonary dysplasia (BPD) remain throughout childhood and can often lead to chronic respiratory diseases in adulthood [5]. Despite advances in treatment, many paediatric respiratory diseases remain incurable, contributing to the increasing burden of noncommunicable respiratory disease in adults globally [1]. The use of cellular-based therapies for the treatment of lung disease has increased in recent years, owing to their pleiotropic effects for amelioration of the main pathologies of lung disease including immune dysregulation and aberrant tissue repair [6]. The most recent focus of clinical studies for the treatment of respiratory disease with cellular therapies is on the adult population, with paediatric patients excluded. These studies have provided a basis for the research in paediatric respiratory disease; despite this, there remains great disparity in clinical trials in paediatric patients. The direct cost of patient treatment for respiratory disease in the EU is estimated at EUR 55 billion annually, and the disability adjusted life years (DALYs), estimated at EUR 280 billion annually [7]. Cellular therapies offer a potential cure for respiratory disease that could prevent their progression into adult respiratory disease, thus reducing the economic burden of treatment throughout a patient’s lifetime.

### 1.1. Cellular Therapy

Cellular therapy is a field in regenerative medicine for the repair, replacement and rejuvenation of native cells or tissues in response to aging, injury or disease [8]. Cellular therapy is considered a multimodal therapy with potential for structural and functional engraftment of cells and immunoregulation [9]. Cellular therapies use both autologous or allogeneic stem cells that are harvested and expanded ex vivo to clinically relevant numbers for transplant to a patient. Stem cells are undifferentiated cells capable of self-renewal that can differentiate into an adult cell [10]. Stem cells have been used both alone and in combination with biomaterials to aid in engraftment by providing a scaffold that supports their growth and aims to prevent migration to other tissues [10]. The use of autologous stem cells for cell and tissue replacement overcomes the limitations of donor supply and potential immune reaction whilst also providing a personalised therapy for patients [8], while allogeneic stem cells can be used to produce an off-the-shelf therapy for patients whose own cells have limited proliferation capacity or who are suffering from a rapidly progressing disease that cannot wait for an autologous therapy to be developed [11].

Stem cells can either be administered in their undifferentiated state or differentiated to a specific cell type as a means of improving their functionality [8]. The use of culturally expanded terminally differentiated adult cells is limited by their ability for expansion, difficulty to culture ex vivo and donor site morbidity upon harvest; owing to this, stem cells are most commonly used for cell-based therapies [12]. However, the first FDA-approved, cell-based orthopaedic therapy was an autologous chondrocyte-based therapy known as Carticel [8]. Stem cells have been isolated from a number of sources including embryonic stem cells isolated from the inner cell mass of the blastocyst, adult stem cells such as hematopoietic stem cells isolated from the bone marrow, adipose derived stem cells, umbilical cord blood derived stem cells and induced pluripotent stem cells (IPSCs) derived from reprogramming of adult somatic cells [13,14]. Strategies to license cells including culture in growth factors and cytokines have also been employed to improve their survival in vivo and alter their modality in vivo by altering their paracrine expression [15,16]. Cell therapy research has been progressing rapidly in recent years, since 2014, when the first stem cell-based therapy, an orphan medicinal product, Holoclar, for the treatment of keratitis caused by limbic deficiency, was approved by the European Medicines Agency [17]. Cellular therapy shows promise as a potential cure for debilitating disease where current treatment options are used for symptomatic management and have no effect on disease progression.

### 1.2. Induced Pluripotent Stem Cells

IPSCs were first successfully reprogrammed from human adult somatic cells in 2007 using factors known to maintain pluripotency in embryonic stem cells [14]. Pluripotent stem cells are capable of differentiating into all lineages of the three germ layers of the embryo [18]. IPSCs have been used in place of embryonic stem cells due to the ethical and safety concerns surrounding their use [19]. IPSCs have shown great potential for disease modelling of patient-specific defects for neural and lung disease [19]. Patient somatic cells carry their exact genetic defect and have been used successfully for the characterisation of the disease phenotype and to determine aetiology. IPSC-derived lung organoids have demonstrated that the major disease phenotype in surfactant B disorders is a lack of lamellar cell bodies [19]. IPSCs have also shown great potential for use in drug screening of multiple novel targets by providing suitable models of the human disease [19]. These models provide relevant preclinical data of drug safety and efficacy that may not be emulated in animal models [19]. IPSCs have also been used in preclinical models for monogenic diseases in combination with gene editing technologies, such as CRISPR/CAS 9, to modify the genetic defect. Schwank and colleagues developed intestinal organoids from paediatric patients with the ΔF508 cystic fibrosis transmembrane conductance regulator gene (CTFR) mutation; using CRISPR/CAS9, they inserted a wild type functional CTFR gene and observed CTFR restoration determined by use of the forskolin assay [20]. The development of functional lung tissue with IPSCs requires induction at the stages of embryonic lung development. Recently, lung organoids containing functional lung alveolar type 2 (AEC2) epithelial cells have been developed from IPSC differentiated NKX2.1+ lung progenitor cells cultured in differentiation medium supplemented with glycogen synthase kinase 3 inhibitor (CHIR9902) and recombinant human keratinocyte growth factor (rhKGF) [21]. Although this study shows promise for lung regeneration, the lung is incredibly complex and is comprised of multiple cell types—therefore, the ability of IPSCs for whole-lung regeneration is currently highly unlikely [22]. Furthermore, IPSCs have potential for ectopic tumour formation, which may account for why other stem cell types have been studied more extensively for the development of cellular therapies.

### 1.3. Mesenchymal Stromal Cells

MSCs are one of the most studied cell types for use in the cell therapy field, owing to their potential mechanisms of action. MSCs are multipotent adult stem cells capable of self-renewal and differentiation into cells of the mesodermal lineage including adipocytes, osteocytes and chondrocytes [13]. They have been found in a number of body tissues including the bone marrow, adipose tissue and umbilical cord blood [23]. The exact mechanism of action of MSCs in vivo are complex, due to their ability to differentiate; initially, they were thought to aid in direct cell replacement. However, a previous study found that differentiation in vivo was relatively low. In vivo, it appears their effects are, in fact, through their paracrine mechanism of action. MSCs have been shown to express growth factors that stimulate endogenous repair pathways [13] and home to sites of inflammation expressing anti-inflammatory cytokines to dampen the host immune response [24]. The ease of isolation, culture and proposed mechanisms of action of MSCs have led to extensive preclinical and clinical research. A randomised, double-blinded phase 1 trial in patients with chronic obstructive pulmonary disease (COPD) recorded no adverse events, confirming the safety of MSCs for patient use [25]. Levels of circulating C-reactive protein (CRP) were shown to decrease following treatment, confirming the potential anti-inflammatory effects of MSCs for treatment of COPD [25].

### 1.4. Preconditioning

Methods have been employed to potentiate the survival and boost the paracrine effects of MSCs in vivo. These methods include culturing MSCs in sublethal hypoxic, proinflammatory, serum starved, oxidative and heat shock environments to license them for their prospective in vivo environment [26]. Hypoxic conditioning of MSCs has been shown to promote expression of cryoprotective, antiapoptotic and proangiogenic factors [27]. In a bleomycin-induced model of pulmonary fibrosis, administration of hypoxia preconditioned bone marrow-derived MSCs (BMMSCs) showed significant reduction in levels of proinflammatory cytokines IL-6 and IL-1β compared to normoxia control MSCs [27]. Expression of the fibrotic mediators collagen type III and connective tissue growth factor (CTGF) were also reduced following treatment with hypoxia conditioned MSCs compared with controls [27]. Preconditioning with proinflammatory cytokines such as (IFN)-γ, IL-6 and TNF-α expressed by neutrophils aims to improve the potency of MSCs through activation of the NF-κB pathway to stimulate expression of anti-inflammatory cytokines [28]. Acute respiratory distress syndrome (ARDS) is primarily mediated by inflammation. To determine the MSC phenotype in the treatment of ARDS, serum from adult patients high in proinflammatory IL-8, IL-6 and IL-10 was pooled and used for their culture [29]. The optimal serum concentration of 0.5% ARDS serum significantly increased expression of anti-inflammatory IL-10 and interleukin receptor agonist (IL-RN), correlating with a decrease in expression of IL-6, IL-1α, IL-8, IL-1β and IFN-γ [29]. Preconditioning of MSCs in ARDS patient serum provides a direct representation of the MSCs phenotype and potential for treatment of the disease.

### 1.5. Extracellular Vesicles (EVs)

The MSC secretome has been investigated as a cell-free therapy boasting the paracrine effects of the cell without the potential tumorigenic effects associated with the cells’ ability to self-replicate [30]. The benefits of developing a cell-free therapy are primarily offsetting the risks associated with cell transplant including potential immune response, formation of an embolism in the patient and preventing the transmission of undetectable viruses [31]. A major barrier in receiving marketing authorisation for cellular therapies is the regulatory criteria they must meet. Conditioned medium and EVs may be regulated similar to a drug product, where potency and dosage can be measured [31]. Large-scale production of secretome products can be carried out using cell lines, limiting the need for donors. Cellular therapies require production of large cell numbers, often lost within the first few days of transplant. Developing secretome products is a more cost-effective process [31]. The MSC secretome is comprised of soluble factors and extracellular vesicles including exosomes and microvesicles [32]. Exosomes are relatively small, between 50 and 200 nm in diameter, and are released by fusion of the mature endosome with the cell surface [32]. Microvesicles are larger at >200 nm in diameter and are released by shedding of the plasma membrane [32]. EVs are shed into circulation under normal physiological conditions and in disease states [30]. Proteins including miRNAs, growth factors, cytokines and mitochondria released by MSCs are packaged in these EVs; their expression indicates their cell origin and the cell’s culture conditions and provides a direct representation of the cells external environment [30]. Preconditioning of MSCs to in vivo disease conditions alters their secretome, resulting in EVs providing the clinical benefits of the cell, easily isolated from conditioned medium by centrifugation for direct administration to patients [30]. In an *Escherichia Coli* (*E. coli*) endotoxin-induced model of acute lung injury (ALI), intratracheal instillation of MSC EVs through the jugular vein lead to a reduction in inflammatory cell influx, oedema, blood and thickening of the interstitium in the lungs of ALI mice models [33]. ALI is acute inflammation that causes disruption to the lung epithelial and endothelial barriers following injury or infection characterised by immediate onset of hypoxemia in the presence of diffuse pulmonary infiltrates [34].

### 1.6. Conditioned Medium

Harnessing the MSC secretome through MSC conditioned medium may encapsulate the full therapeutic benefit of the cell including both soluble factors and EVs [35]. MSC conditioned medium consists of all expressed cytokines, proteins, growth factors and EVs of the cell [36]. In a comparative study for the evaluation of MSC conditioned medium and the MSCs as a therapy both alone and in combination, comparable results for efficacy were recorded [37]. In a murine model of *E. coli*-induced ALI, MSC-derived conditioned medium administration led to a reduction in septal thickening, alveolar haemorrhage, alveolar infiltrates and fibrin strands determined by histological staining of the lung when compared to untreated controls [38]. The effect of MSC conditioned medium and the MSCs, alone, demonstrated a mirrored reduction of neutrophil influx and lung permeability [38]. In a bleomycin-induced model of pulmonary fibrosis in Wistar rats, treatment with adipose-derived MSC conditioned medium lead to a reduction in collagen deposition associated with fibrosis when compared to untreated controls [39]. The development of pulmonary hypertension, right ventricular hypertrophy and associated cardiac dysfunction were significantly reduced in animals treated with conditioned medium when compared to controls [39]. The MSC secretome shows great potential as a cell free therapy; however, proteomic analysis of different MSC sources has shown varying expression profiles. The development of a cell-free therapy will require determination of the most beneficial cell source to ensure the highest therapeutic potential.

## 2. Respiratory Diseases

### 2.1. Bronchopulmonary Dysplasia

BPD is a chronic respiratory disease of preterm infants resulting from lung immaturity as a consequence of interruption of lung development occurring in the final weeks of gestation [4]. It was originally diagnosed in 1967 [40] as pulmonary fibrosis and airway smooth muscle hypertrophy as a result of prolonged mechanical ventilation leading to respiratory decline and ventilator dependence [4]. Advances in treatment with surfactants and controlled ventilation strategies have improved survival rates and have led to the determination of a new clinical form of BPD [4] defined as a greater than 21% oxygen dependency for more than 28 days determined at 36 weeks of gestation [41]. In Europe, the incidence of BPD in preterm infants below 30 weeks gestation is over 30% [41]. BPD is characterised by an arrest of lung development in the saccular stage leading to alveolar and vascular simplification and diffuse pulmonary inflammation induced by mechanical ventilation [4,41]. Current treatments using corticosteroids and surfactants enhance lung compliance but also lead to an arrested alveolar development. The lungs are incapable of adjusting to increased respiratory requirements, leading to a requirement for mechanical ventilation, which, in turn, leads to increased damage through inflammation [4]. The inefficiency of these therapeutic approaches and potential irreversible damage caused has resulted in preclinical and clinical studies using cellular therapies, with a particular focus on MSC based therapies [4]. Resident MSCs in the lung are necessary for lung development through expression of chemokines and growth factors that promote proliferation of lung epithelial and endothelial cells [42]. MSC dysfunctional transdifferentiation and epithelial signalling is common in patients with BPD and is linked to PDGFRα/TGFβ-1/β-catenin signalling dysfunction, leading to impaired alveolarization [43]. An early marker of BPD development is presence of MSCs in tracheal aspirate [43].

Van Haaften and colleagues performed a number of in vitro and in vivo experiments to determine the potential efficacy of using BMMSCs for the treatment of BPD [44]. In vitro BMMSCs were cultured in a modified Boyden chamber with oxygen-deprived lung cells placed in the bottom. BMMSCs were shown to migrate to the damaged lung cells, confirming their potential for homing to damaged tissue. MSCs co-cultured with hyperoxic lung tissue were found to express surfactant C and developed lamellar cell bodies, known immunophenotypic and ultrastructure characteristics of AE2 cells. An in vivo model of BPD was induced by exposure of a rat to hyperoxic conditions (95% O_2_). Histological staining confirmed alveolar simplification a characteristic of BPD. There were two treatment groups: (1) a prevention group treated at P4 and (2) a regeneration group treated at P14. Both received an intratracheal injection of 1.0 × 10^5^ of rat BMMSCS. MSCs injected at P4 were shown to engraft in the lung. Engrafted cells developed an AE2 cell phenotype determined by expression of surfactant C; they were also found to reduce pulmonary tension to levels similar to untreated controls. Furthermore, MSCs injected at P4 were shown to significantly improve alveolarization compared to untreated control models of BPD. Injection of MSCs at P14 did not show a similar significant improvement. This group also performed in vitro studies using BMMSC conditioned medium to test the paracrine mechanism of MSCs. Culture of AEC2s in BMMSC conditioned medium in hyperoxic conditions prevented O_2_-induced apoptosis and DNA damage. A scratch assay was also performed; AEC2s cultured in BMMSC conditioned medium had significantly higher wound repair than AEC2s cultured in DMEM, alone [44].

In another preclinical study, umbilical cord blood-derived MSCs (UBMSCs) were administered intratracheally in a newborn rat model of BPD. BPD was confirmed by decreased lung compliance, alveolar simplification and distal air space enlargement, all known hallmarks of BPD [45]. Animals were selected to receive prophylactic treatment at P4 or regenerative treatment at P14. At P4, animals received an intratracheal injection of 3 × 10^5^ human UBMSCs (hUBMSCs); for regenerative studies, at P14, animals received 6 × 10^5^ hUBMSCs. Prophylactic delivery of MSCs prior to induction of BPD was found to partially preserve alveolar growth, and lung compliance was significantly higher in models treated with UBMSCs. A 6-month-long safety study found that untreated controls had a significantly reduced exercise capacity compared to animals who received UBMSCS. Whole-body CT scans confirmed that MSCs did not induce tumour formation and confirmed that MSCs were safe long-term, up to 6 months. This group also carried out testing using UBMSC conditioned medium due to the low engraftment rate of cells in vivo. Animals receiving prophylactic injections of conditioned medium from P4 to P21 had partial efficacy in preventing arrest of lung angiogenesis and were found to significantly reduce hypertrophy [45]. Long-term safety studies also confirmed the long-term safety of treatment with conditioned medium up to 6 months [45]. BPD is a multifactorial disease caused by immature lung development through alveolar simplification and pulmonary inflammation. Chou and colleagues developed a BPD model of maternal-induced inflammation by intraperitoneal injection of lipopolysaccharide (LPS) from *E. coli* on days 20 and 21 of gestation [46]. At term, upon delivery, rat pups were exposed to hyperoxia (85% O_2_) up to day 14 postdelivery. At postnatal day 5, the rats received human placenta-derived MSCs intratracheally. Rats exposed to hyperoxia treated with MSC had reduced levels of proinflammatory IL-6 and TNF-α compared to untreated controls. Treatment with MSCs was also shown to significantly reduce collagen deposition when compared to untreated controls exposed to hyperoxia. Collagen deposition in MSC-treated animals was comparable to animals exposed to normoxia [46].

Cell-free therapy using exosomes derived from human umbilical cord Wharton’s jelly MSCs (WJMSCs) have also shown great potential for the treatment of BPD. Willis and colleagues developed a mouse model of BPD by exposure to hyperoxia (75% O_2_) from postnatal (PN) day 1–7 [47]. Three treatment groups were established: the first was administered a single dose of WJMSC-derived exosomes; the second, a single dose of BMMSC-derived exosomes; and the third, a single dose of human dermal fibroblast-derived exosomes (vehicle control) at PN day 4. Animals received a single bolus dose of the product of 0.5 × 10^6^ MSCs intravenously based on findings from previous studies. Comparative studies at PN day 14 showed histological staining comparable to development of human BPD with arrested alveolar growth, large airspaces and incomplete alveolar septation. In contrast, both groups treated with MSC-derived exosomes showed functional alveolarization and restoration of the lung infrastructure. A long-term study assessed on PN day 42 confirmed functional alveolarization, and restoration of the lung infrastructure was maintained when compared to untreated controls. Pulmonary testing was also carried out at day 42 to determine the effects of lung architecture remodelling on function. Untreated controls exposed to hyperoxia developed an emphysema phenotype with an associated change in the pressure volume loop and air trapping. Treatment with WJMSC exosomes augmented these changes in the treated group. An in vitro study to determine the effect of MSC exosomes on polarized (M1) bone marrow-derived macrophage phenotype found that WJMSC exosomes dose dependently reduced mRNA expression of proinflammatory genes [47]. This confirmed the potential of exosomes for immunomodulation.

### 2.2. Paediatric Acute Respiratory Distress Syndrome

ARDs is a major cause of mortality worldwide, with estimates of 10 to 86 cases occurring per 100,000 [48]. ARDs is characterised initially by development of capillary congestion, atelectasis, interalveolar haemorrhage and alveolar oedema. After a number of days, a hyaline membrane is formed, epithelial cells become hypertrophic and interstitial oedema develops [48]. ARDS has been recognised in children for a number of years; however, a definition for paediatric ARDS (PARDS) has only been established recently by the Paediatric Acute Lung Injury Consensus Conference (PALICC) [49]. The differences in anatomy and physiology of the respiratory system of paediatric patients including levels of alveolar maturation, chest wall compliance, their respiratory muscle reserve and metabolic demands mean that they do not fall under some of the criteria outlined in the Berlin definition of adult ARDs [49]. The PALICC definition of PARDs uses pulse oxygen (PaO_2_) in place of place of partial pressure of oxygen and stratifies the disease severity using the oxygen index (OI) in place of the ratio of PaO_2_ to fractional concentration of oxygen-inspired air (FiO_2_) used in the Berlin definition of ARDS [50].

PARDs is commonly caused by direct lung injury, most commonly caused by respiratory infection triggered via the respiratory syncytial virus (RSv) [51], but can also be initiated by pneumonia, aspiration of gastric contents and other factors such as near drowning [52]. Indirect injury including sepsis not developed in the lung, nonthoracic trauma and pancreatitis, among others, are also potential instigators [48]. The pathogenesis of ARDs is mainly through inflammatory processes, with initial response to injury resulting in innate immune cell infiltration, causing damage to the endothelial and epithelial barriers and resulting oedema in the alveoli [48]. Recruitment of adaptive immune cells further potentiates this inflammation prior to induction of the secondary proliferative phase of ARDS, where repair takes place [48]. The treatment of ARDS is a multistep process: Initial treatment requires identification of the root cause, followed by a controlled mechanical ventilation regime with low tidal volumes in order to limit further exacerbation of the ALI [50]. Pharmacological treatments have had limited success with only transient improvements in oxygenation in treating PARDs [50]. With increased numbers of patients requiring ventilation, an effective cure for PARDS needs to be developed. Because of this, there is increasing interest in developing cell-based therapies.

ARDS is a severe form of ALI. Preclinical models of ARDS have been developed through development of a ventilation-induced injury by introduction of a bacterial endotoxin. Curley and colleagues exposed anesthetised rats oxygen, causing ventilation induced injury (inspiratory pressure 35 cm H_2_O, respiratory rate 18/min and positive end expiratory pressure 0 cm H_2_O) [53]. ALI was confirmed by a 50% reduction in respiratory compliance. Animals received two doses of MSCs by injection. The first dose was administered directly after ventilation-induced lung injury (VILI), and the second, 24 h later. Assessments carried out at 48 h postinjury found significantly improved arterial oxygenation and significantly restored static compliance compared to controls. Lung wet-to-dry ratios and alveolar protein concentration were also reduced following MSC treatment, confirming that microvascular permeability was restored by treatment with MSCs. MSC treatment was shown to attenuate inflammation by reduction of alveolar expression of TNF α. Bronchoalveolar lavage (BAL) neutrophil counts were significantly reduced following treatment with MSCs, and a significant increase in anti-inflammatory IL-10 expression was also observed following treatment. MSC treatment was also found to significantly reduce alveolar thickening and to restore airspace volume compared to untreated controls [53].

In a study by Lee and colleagues, they developed an ex vivo *E. coli* endotoxin-induced model of pneumonia in a perfused human lung [54]. They used this model of ALI to determine the effects of clinical grade BMMSCs. They found that IV infusion of BMMSCs 1 h following induction of the ALI restored alveolar clearance to normal levels. They also found levels of proinflammatory IL-1β and IL-8 were reduced following treatment, supporting the potential of MSCs to mediate the inflammatory response. They found that MSCs dose dependently decreased bacterial load, doubling of the dosage to 10 × 10^6^ cells per kg of body weight decreased the bacterial load by 40%. They also carried out an in vitro study using alveolar fluid from MSC-treated lungs as conditioned medium for culture of *E. coli*; they found that alveolar fluid from lungs treated with MSCs had increased antimicrobial activity compared to that of untreated lungs [54]. Success in preclinical models led to a multicentre, open-label, dose-escalation phase-one clinical trial using a single dose of allogeneic BMMSCs administered intravenously to nine patients suffering from moderate-to-severe ARDs [NCT01775774] [55]. This trial confirmed the safety of delivery of allogeneic MSCs to patients suffering from moderate-to-severe ARDs, and no serious adverse events were found as a result of administration. MSCs were found to dose dependently improve the mean lung injury score, with patients receiving the highest dose of 10 million cells per kg of body weight having the highest reduction. Two of nine patients were extubated prior to day three, and none of the patients required nitric oxide orbronchodilators for treatment of refractory hypoxemia [55]. A follow-up phase 2A [NCT02097641] double-blinded, randomised placebo trial was carried out to assess safety in a larger population of 63 patients using the highest dose of 10 × 10^6^ cells/kg of body weight [56]. The trial confirmed there were no adverse infusion-related or respiratory events following treatment with MSCs. However, this trial lacked statistical power, and this group is currently recruiting for a phase 2B trial [NCT03818854] to determine the efficacy of their MSC product in a larger sample population [56]. The efficacy of MSC treatment for patients with ARDs needs to be confirmed. These preclinical and clinical studies confirm their potential for adult patients—inclusion of paediatric patients in further trials is needed.

### 2.3. Asthma

Asthma is one of the most common noncommunicable respiratory diseases globally [1]: it is estimated to affect over 300 million people, with its incidence projected to increase by 30% by 2025 [57]. Paediatric asthma is one of the most common chronic diseases of childhood, with its high prevalence being a major cause of disability globally [58]. Asthma is classified as a heterogenous disease characterised by airway hyperresponsiveness, mucus secretion, varying levels of bronchoconstriction and chronic inflammation of the airways and lungs [59]. Over half of paediatric asthma sufferers are diagnosed by the age of three; however, diagnosis is somewhat difficult, as symptoms such as wheezing are common in paediatrics, and pulmonary testing, including spirometry, is difficult in patients under the age of 5 [60]. The main criteria for diagnosis are symptoms such as wheezing, airway hyperresponsiveness and airway obstruction that show response to bronchodilators, together with risk factors including familial predisposition as well as atopy [60]. Diagnosis in older children can be determined using lung volumes by plethysmography to determine if there is air trapping and hyperinflation, which occurs with airway obstruction [60]. The prevalence of asthma in childhood is higher in males than females; however, an increased prevalence in older female children can be seen after puberty [60]. The development of paediatric asthma is linked to exposure to environmental aeroallergens early in an infant’s life, leading to airway sensitization. Recurring wheezing due to bacterial and viral infections is also another risk factor for asthma development [60].

The main pathological feature of asthma is inflammation mediated by immune cells in the airways producing proinflammatory cytokines, recruiting both innate and adaptive effector cells, leading to chronic inflammation [59]. Chronic inflammation mediates remodelling of the airways including epithelial injury, remodelling of the basement membrane, changes in volume of airways smooth muscle and increased angiogenesis and goblet cell metaplasia [59]. Severe asthma with frequent exacerbations is often unresponsive to current pharmacological treatments and is associated with a higher mortality and lower quality of life in patients compared to milder forms of the disease [59]. The use of biologic treatments such as the immunoglobulin E (IgE) monoclonal antibody omalizumab have shown promise in minimising exacerbations in several forms of the disease [59]. In Europe, omalizumab is recommended as an add-on treatment limited for use in paediatric patients over the age of 6 [61]. Long-term dosage has shown some clinical efficacy; however, repeated treatment is required. Cellular therapy may provide a more effective treatment through anti-inflammatory pathways and through potential repair or rejuvenation of damaged lung tissues. Trzil and colleagues developed a feline model of chronic allergic asthma by sensitisation to Bermuda grass antigen (BGA) to study the long-term effects of MSC therapy [62]. Intradermal testing and bronchoalveolar lavage fluid (BALF) analysis were carried out to confirm that the animals did not have a pre-existing allergy to BGA. Sensitisation to BGA was carried out over a 28-day period by injection of the allergen at day 0 and day 21 and intranasal delivery at day 14. Asthmatic phenotype was confirmed by greater than 17% neutrophils in BALF. Animals were exposed to BGA weekly or bimonthly over a 9-month period to simulate the inflammation and airway remodelling seen in chronic allergic asthma. Treated animals received a dosage of between 3.64 × 10^6^ and 2.5 × 10^7^ of allogeneic adipose derived MSCs from healthy donor felines intravenously through a cephalic catheter bimonthly for 2.5 months. Airway remodelling was monitored by CT scan at 8 months and 10 months after initial treatment with MSCs; there was a significant decrease in global lung attenuation and bronchial thickness score at month 8 from the treated group in comparison with the placebo. Results at month 12 did not show a significant difference, possibly indicating a repeated infusion was required [62]. These results indicated that further studies with a particular focus on dosage were required but confirmed the safety of using MSCs long-term for the treatment of chronic asthma.

Following from this study, Ahmadi and colleagues carried out an in vivo experiment in mice to determine the effects of rat BMMSCs and rat BMMSC conditioned medium on an ovalbumin-induced rat model of chronic asthma [63]. Rats were exposed to ovalbumin for 32 days to sensitize them. Following development of sensitization, the rats were separated into three groups: the first was treated with BMMSCs, the second was treated with BMMSCs conditioned medium and the third group served as a control. Development of an asthmatic phenotype by sensitization was confirmed using methacholine, traditionally used in the diagnosis of asthma. On day 33, one day post sensitisation, rats were administered 2 × 10^6^ rat BMMSCs or 50 μL of conditioned media by intratracheal administration. Two weeks after treatment, animals were anaesthetised and their trachea removed to determine tracheal response to methacholine. Tracheal response was improved in animals that were treated with BMMSCS and BMMSCs conditioned medium, confirmed by a significant decrease in methacholine E50 in tracheal pieces compared to sensitised controls. Levels of white blood cells were increased in all sensitised rats when compared to healthy controls, percentage of neutrophils and eosinophils were significantly lower in BMMSC and BMMSC conditioned medium-treated animals compared with sensitised control animals. Levels of CD3+ CD4+ and CD3+ CD8+ lymphocytes were significantly lower following sensitisation. Treatment with BMMSCs and BMMSC conditioned medium significantly increased levels of CD3+ CD4+, corresponding with a simultaneous decrease in CD3+ CD8+ lymphocytes. Treatment with MSC showed a significantly greater increase in CD3+ CD4+ compared with treatment with conditioned medium. Pathological remodelling of lung tissue seen in asthma including emphysema, atelectasis, hyperaemia, epithelialisation and leukocyte infiltration were significantly higher in sensitized groups compared to healthy controls [63]. Treatment with BMMSCs and conditioned medium lead to a significant decrease in the pathological changes compared to the sensitised control. Treatment with MSCs showed a greater decrease in pathological changes than treatment with conditioned medium, alone [63]. Another study carried out by McCarthy and colleagues confirmed the effectiveness of using conditioned media from both BMSCs and UBMSCs on E.coli, Staphylococcus aureus and Klebsiella pneumoniae isolates [64]. Conditioned medium was passed through a vibrating mesh nebuliser, and clinical isolates from patients were added to determine the antimicrobial properties of the medium. Pre- and postnebulisation, BMMSC conditioned media were shown to significantly reduce proliferation of Staphylococcus aureus and Klebsiella pneumoniae but had a lesser effect on *E. coli* isolates. This study confirmed the antimicrobial properties of MSC conditioned medium on clinical isolates including antibiotic-resistant Klebsiella pneumoniae following delivery via aerosol [64]. This further confirms the potential of MSC-derived conditioned media as a treatment in the lung.

Kang and colleagues also carried out a study to determine the effects of hUBMSCs in an ovalbumin-induced murine model of asthma [65]. Six-week-old mice were sensitised with ovalbumin by intraperitoneal injection at day 1 and day 14. Following sensitisation, animals received a dose of 1 × 10^6^ hUBMSCs via the tail vein. On days 29, 30 and 31, mice were challenged by intranasal administration of ovalbumin. Following the last ovalbumin challenge, methacholine was used to determine the airway hyperresponsiveness, with sensitised animals having a significant increase in airway hyperresponsiveness when compared to untreated control animals. Animals treated with hUBMSCs, however, showed a significant decrease in development of airway hyperresponsiveness. Sensitised animals had increased infiltration of inflammatory cells including eosinophils, macrophages, neutrophils and lymphocytes. Treatment with hUBMSCs was shown to attenuate this inflammation with an increased reduction in eosinophilic infiltration when compared to untreated sensitised controls. Furthermore, histopathological features of the asthma phenotype including the basement membrane, epithelium and subepithelial smooth muscle layer thickening of goblet cells was ameliorated by treatment with hUBMSCs when compared to untreated controls. Levels of IL-4, IL-5 and IL-13 mRNA expression were significantly reduced in BAL following treatment with hUBMSC, and levels of anti-inflammatory IL-10 and TGF-β were significantly increased following treatment when compared to sensitised controls. Levels of the T regulatory cells f CD4+ CD25+ Foxp3+ in the draining lymph nodes and spleen were significantly higher following treatment when compared to untreated controls [65]. These results confirm the potential of MSCs in vivo for mediation of chronic inflammation seen in asthma. Clinical studies are needed to confirm their safety, efficacy and optimum dosage for the treatment of patients.

### 2.4. Cystic Fibrosis

Cystic fibrosis is an autosomal recessive genetic disease cause by mutation in the CF transmembrane conductance regulator gene (CTFR) [66]. In the past, the incidence of CF globally has been estimated in epidemiological studies, but more recently, newborn screening has been the major focus, owing to the varying incidence from country to country. In Europe, CF is estimated to affect, on average, 1:3500 live births [3]. The adult population affected by CF is increasing and is estimated to be 40% of the population by 2025 [66]. The CTFR gene is responsible for transport of chloride and bicarbonate. Its dysfunction is linked to impaired mucociliary clearance as a result of abnormal hydration of airway mucus [66]. Although CF predominantly affects the lungs, it is classified as a multiorgan disease also affecting the gastrointestinal tract, liver and the pancreas [66]. CF is commonly caused by mutations in the ΔF508 gene; however, over 2000 mutations have been linked to the development of cystic fibrosis [66]. Chronic inflammation is the predominant pathological feature of CF. One of the major clinical symptoms, bronchiectasis, develops as a result of free neutrophil elastase activity [66]. This neutrophil protease exacerbates the innate immune response in the lung leading to increased mucus production and damage to the lung epithelium [66]. Further exacerbation of inflammation occurs due to development of lower respiratory tract infections, resulting from increased susceptibility to Gram negative bacteria as a result of bronchiectasis [66]. Pseudomonas aeruginosa is the most common cause of respiratory infection, leading to increased damage to the airways [66]. CF was originally considered a paediatric disease. Advances in care have led to its development to an adult disease [66]. The median life span for patients suffering from CF has increased from a few months to a median of 40 years [66]. Recent studies have linked increased susceptibility to lower respiratory tract infection to decreased lung function in CF patients as a result of bronchiectasis [67]. Development of bronchiectasis has increasing prevalence from 29.3% at 3 months old to 61.5% at 3 years of age [68]. The data indicates the need for targeted therapy at the developing stages of the disease in infants to maintain lung function and prevent further development of the disease. The development of the adult disease is linked to newborn screening, with early diagnosis and better treatment options leading to increased survival of patients to adulthood [66]. The need for disease treatment in the adult population is clear, with its increasing prevalence a major cause for concern. However, an effective treatment in infancy to ameliorate or even prevent disease progression could potentially eliminate the adult disease. Cellular therapies are being researched as a potential cure, with IPSCs being a major focus of current research.

Firth and colleagues generated IPSCs from patients homozygous for the deletion of the ΔF508 CTFR gene mutation and used CRISPR CAS9 gene editing to insert a functional CTFR gene [69]. Donor fibroblasts were reprogrammed using either a six-factor polycistronic lentivirus followed by excision using Cre-recombinase or using a sendai virus with four individual reprogramming factors. To insert a functional CTFR gene, a custom CRISPR system was developed. This system consisted of a plasmid encoding the Cas 9 protein and a codon under the control of the eukaryotic transcription elongation factor 1 alpha 1 (EEF1A1) for expression in human cells. The donor vector with corrected CTFR sequence had a GRP-Puro-TK casette inserted to allow for identification of the corrected IPSCs using puromycin. Differentiation to lung epithelial cells took place over 48 days. Cells were driven through definitive endoderm and anterior foregut endoderm to NKX2.1+ lung progenitor cells, followed by maturation to lung epithelial cells. CTFR current analysis by patch clamp method was carried out to confirm functional CTFR correction. Cells were introduced to a cocktail of forskolin, genistein and isobutyl methyl xanthine (IBMX) to determine the effect on CTFR current. Half of corrected cells showed an increase in CTFR current similar to wild type CTFR controls. A CTFR inhibitor was used to determine if the increased current was a result of CTFR production, which was confirmed. Mutant untreated cells showed no response to the cocktail [69].

Another study carried out by Crane and colleagues used zinc finger nucleases to carry out homology-directed repair of the mutations of the endogenous CTFR gene in CF patient-derived IPSCs [70]. Patient fibroblasts containing either ΔI507 or ΔF508 deletions were reprogrammed using retroviral vectors expressing the reprogramming factors OCT4, SOX2, KLF4, Nanog and C-MYC. This group used zinc finger nucleases that targeted CTFR exon 10, thatrecognised DNA sequences upstream of the ΔI507 or ΔF508 deletions, to allow them to target either mutation. Corrected IPSCs clones were screened using real-time polymerase chain reaction (RT-PCR) and sequenced to confirm corrected CTFR expression. CTFR expression was confirmed for both the ΔI507 and ΔF508 alleles. Cells were then differentiated through definitive endoderm and anterior foregut endoderm to NKX2.1+ lung progenitors to lung epithelial cells over 19 days. To determine if the corrected IPSCs had a functional restoration of CTFR chloride channel activity, differentiated epithelial cells were grown on permeable supports to confluent mono layers. Ussing chamber analysis was used to assess this. Introduction of forskolin and genistein stimulated a CFTR-dependent short-circuit current in corrected cells but not in controls. When compared with control WA09 human embryonic stem cells corrected epithelial cell functionedin a similar manner, and addition of VX809, a CTFR modulator, did not increase CTFR expression [70]. These studies confirm the potential of genetically modifying patient-specific IPSCs, which would allow for a more personalised therapy; however, further studies confirming the engraftment of these cells with functional CTFR expression in vivo are required.

A recent study by Villamizar and colleagues was carried out to determine the potential of MS-derived exosomes for delivery of zinc finger activators targeted to the CFTR gene promoter (CFZF-VPR) [71]. Human BMMSCs (6 × 10^6^) were co-transfected with CFZP-VPR and Connexin 43 (Cx43), and after 48 h, exosomes were harvested from the medium. Uptake of MSC-derived exosomes was confirmed by labelling them with BODIPY and culturing them with human bronchial epithelial cells (HuBEC) from patients homologous for the ΔF508 CTFR mutation. Light microscopy immunofluorescence confirmed the presence of BODIPY staining of the cells. Furthermore, expression of CFZF-VPR in the ΔF508 CTFR HuBECs was confirmed by quantitative RT-PCR (qrt-PCR). To determine if MSC-derived exosomes expressing CFZF-VPR could induce CTFR expression, wild type HuBECs’ and ΔF508 CTFR HuBECs’ expression of CTFR was measured following treatment with exosomes. Increased levels of CTFR were confirmed in both cell types, confirming the ability for the exosomes to induce CTFR expression. To determine if the increased CTFR expression had any functional significance, they performed a halide assay, where the cells were modified to express iodide-sensitive yellow fluorescent protein (YFP). They replaced the chloride ions in the cells with iodide ions and incubated them with forskolin to stimulator CTFR prior to further culture in the presence of the CTFR potentiator (VX770) and corrector (VX809). In both wild type HuBECs and ΔF508 CTFR HuBECs that had been treated with CFZF-VPR exosomes, there was a significant decrease in fluorescence compared to untreated controls [71]. This confirmed the ability for MSC CFZF-VPR exosomes to be used to provide a functional repair of the CTFR mutation in HuBEC cells.

### 2.5. Interstitial Lung Disease

Childhood interstitial lung disease (chILD) is a heterogenous group of over 200 chronic lung diseases characterised by abnormal pathology of the lung interstitium and distal air spaces, resulting in abnormal gas exchange [72,73]. chILD is quite a rare disease; however, its global incidence is not clearly identified, with the incidence in the UK and Ireland estimated at 3.6 per 1 million [74]; in Australia, estimates are 1·5–3·8 per 1 million [75], and in Germany, it is estimated at 0·13 per 100,000 children per year [76]. The ability for clinicians to diagnose the disease is impaired due to the overlapping symptoms with more common lung diseases [77]. Disease phenotype is quite varied for this group of diseases. Common presentations include persistent tachypnoea, inconsistent cough, a wheeze on auscultation, acute respiratory infection induced respiratory morbidity that is unexpected, borderline oxygen saturation and failure to thrive [77,78]. These symptoms are not enough to diagnose the disease—clinical manifestations including chest deformity, clubbing and hypoxemia are considerations. Familial history, a physical examination with pulmonary testing, chest x-rays and genetic testing must all be carried out for diagnosis [77]. Common diseases in infants include lung developmental diseases such as pulmonary hypoplasia, alveolar dysplasia and pulmonary interstitial glycogenosis genetic surfactant disorders caused by mutations of the SP-B, SP-C and ABCA3 genes [79]. In children, presentations include exposure-induced disease such as hypersensitivity pneumonitis, radiation pneumonitis, fibrosis; lymphatic and vascular diseases including pulmonary lymphangiectasia or pulmonary capillaritis; and connective tissue diseases including systemic juvenile idiopathic arthritis, dermatomyositis and polymyositis [77,80]. Manifestations and pathogenesis of the disease in infancy, childhood and adulthood all vary; despite this, the primary focus of research has been on the adult population [78]. The pathogenesis of ILD is quite complex. Although an initial inflammatory response is thought to occur, anti-inflammatory therapies have shown little effect in altering disease progression [80]. It is thought that aberrant wound healing following lung injury is the major pathological feature [80]. Although chILD is considered an orphan disease owing to its low incidence, the disparity in symptoms and pathogenic phenotypes between infants and children, together with lack of knowledge, may be resulting in underestimated disease figures [77]. Furthermore, the cost of patient care into adulthood and patient’s quality of life are affected by a lack of efficient treatment strategies. There is a clear need for research to expand to the paediatric population.

Thus far, the studies highlighted in this review have predominantly focused on the anti-inflammatory effects of MSCs; however, MSCs have also shown great promise for wound repair. Previous studies have found that MSCs can accelerate healing of cutaneous wounds through improved re-epithelisation by modulating inflammation and through promoting angiogenesis and granulation tissue formation, thus regulating extracellular matrix remodelling [81]. A study by Akram and colleagues was carried out to determine the potential paracrine mechanism of MSCs and their conditioned medium for repair of lung epithelial cells [82]. In this study, an epithelial cell line A549 cells (AEC) and primary human small airway epithelial cells (SAEC) were grown in mono layer for an in vitro 3D scratch assay. To determine if Human BMMSCs could migrate to sites of injury, MSCs labelled with DiO (3,3′-dioctadecyloxacarbocyanine perchlorate) were co-cultured with labelled AEC cells. AECs were found to migrate directly into the wound and to close the gap and were significantly higher than controls not cultured with MSCs. Mass spectrometry confirmed MSCs secreted fibronectin, lumican, periostin and insulin-like growth factor binding protein-7 (IGFBP-7) factors implicated in wound repair. Fibronectin and lumican were shown to improve SAEC wound repair in the absence of serum supplementation but required serum supplementation to induce repair of AEC cell line. Periostin was capable of inducing wound repair in both AEC cells and SAEC cells in serum-free medium, and IGFP-7 was capable of stimulating wound repair of SAEC cells but not AECs. These results confirm MSCs secrete factors that promote wound repair; however, the effectiveness may be determined by the cell type. As aberrant AEC migration is a feature in developing fibrosis, the effectiveness of these MSC secretome factors in inducing AEC migration was determined by collagen drop assay. AECs were suspended in a collagen droplet supplemented with 0.2% serum and dropped onto a surface coated with fibronectin, lumican or periostin; both lumican and fibronectin were capable of stimulating migration at an optimal concentration at 10 ng/mL [82]. There is great potential for MSCs in the repair of lung cells.

A common form of adult interstitial lung disease is idiopathic pulmonary fibrosis; in children, however, pulmonary fibrosis (PF) is uncommon, with delayed diagnosis or misdiagnosis a potential contributory factor to it poor prognosis [73]. CT scans of paediatric patients with PF often consist of prominent ground glass opacification that progresses to cystic changes with reticular abnormalities, which do not replicate the classic findings in adult patients, including honeycombing and traction bronchiectasis [73]. Despite this, fibrosis occurs, although not with the classic pathophysiology seen in idiopathic fibrosis. Most often, fibrosis in children has been seen in surfactant disorders, on radiation- or chemotherapeutic-induced injury and in rheumatologic disease [73]. MSCs have shown to be effective in traditional bleomycin-induced preclinical models of fibrosis [83]. Reddy and colleagues administered bleomycin via intratracheal injection and followed up with delivery of 40 × 10^6^ adipose tissue derived MSCs (ADMSCs) at day 3, 6 and 9 via a tail vein injection postbleomycin introduction [83]. Survivability was significantly increased following treatment with ADMSCs compared to both untreated controls and animals treated with pirfenidone, an FDA-approved drug for fibrosis that inhibits TGF-β-induced collagen synthesis. Bleomycin injury in control animals presented as a significant increase in collaged deposition resulting in scar formation. Treatment with ADMSCs significantly ameliorated this collagen deposition and scar formation, as evidenced by significant reduction in lung weights. Histological staining revealed distinct pathological features of fibrosis in untreated controls. ADMSCs ameliorated these changes with maintenance of alveolar structure. Ashcroft scores for ADMSC-treated and pirfenidone-treated animals were significantly reduced when compared to untreated controls. ADMSCs were shown to downregulate expression of matrix metalloproteinases, reducing development of fibrosis [83]. Success in preclinical models has led to clinical studies that have also reported success. A phase 1b trial to study the safety and efficacy of endobronchial infusion of autologous ADMSCs confirmed the safety of using ADMSCs to treat idiopathic pulmonary fibrosis [84]. Patients received separate doses of 0.5 × 10^6^ ADSCs per kg of body weight via a catheter through the bronchoscopic channel. Although no statistically significant differences were met, no adverse events were recorded [84]. Owing to the rarity of pulmonary fibrosis in children, diagnosis can often be delayed until a chronic fibrosis is present. MSCs offer a potential repair of damage tissues, together with antifibrotic properties to prevent further disease progression. Furthermore, treatment of paediatric patients may offer a prophylactic treatment to the development of pulmonary fibrosis into adulthood. Increased incidence of fibrosis has been seen as we age, linked to decreased telomerase activity and stem cell impairment, which is also why disease phenotype in children is so difficult to diagnose [85].

Surfactant disorders are another common group of paediatric interstitial lung diseases that have been targeted using IPSCs. Leibel and colleagues developed 3D lung organoids from patients with surfactant disorder B-derived IPSCs [86]. Fibroblasts from patients with the surfactant B p.Pro133GlnfsTer95 (PRO133) gene mutation were reprogrammed with the transcription factors OCT4, SOX2, KLF4 and C-MYC using a sendai virus system. The resulting IPSCs were then reprogrammed with a functional wild type surfactant B cDNA using a lentiviral vector with a SV40 promoter driving eGFP-IRES-puromycin to identify corrected IPSCS. IPSCS were differentiated to lung progenitor cells on Matrigel in monolayer for 11 days before 3D culture to organoids using a 3 steps differential medium process for an additional 40 days. The lung organoids were developed in Matrigel containing transwell inserts to mimic foetal lung development, using inputs required for branching morphogeneisis in particular. Levels of SFTPB gene expression were compared in untreated patient derived IPSCs, IPSCs with corrected SFTPB gene (hiPro133 + SFTPB-GFP iPSC) and a control IPSC line. Corrected IPSCs had a significantly higher SFTPB expression than both control IPSC lines. Following 3D differentiation, organoid epithelial and mesenchyme markers of the proximal and distal lung were measured. hiPro133 + SFTPB-GFP IPSCs showed positive expression of SFTPB and mature SFTPC, unlike uncorrected patient-derived IPSCs. Corrected IPSCs also showed positive staining for the alveolar type 2 cell marker HTII-280 and the alveolar type 1 cell marker podoplanin (PDPN). Using transmission electron microscopy, alveolar type 2 cells of lung organoids were examined to determine if lamellar cell bodies were present. hiPro133 + SFTPB-GFP iPSC had organised cystolic lamellar cell bodies with microvilli at the apical cell membranes, tubular myelin surrounding the cells and distinct tight junctions between cells. There were no lamellar cell bodies in uncorrected patient IPSC-derived lung organoids, confirming that a functional restoration can be achieved using lentiviral vectors [86]. This study is promising; however, the correction of the mutation is prior to development of lung progenitor cells and lung organoids. To confirm the potential, this study would need to be repeated in vivo in an animal model with genetic surfactant B deficiency.

## 3. Clinical Studies

Success in preclinical studies for BPD has led to progression to clinical trials to determine the safety and efficacy of MSC delivery, predominantly umbilical cord-derived MSCs, and their exosomes for potential treatment. As BPD is limited to infants, the trials listed below in Table 1 are restricted to paediatric participants. The majority of current trials are restricted to adult patients, further confirming the disparity in the current treatment approach and the need for clinical research to progress to paediatrics. A search on clinical trials.gov revealed the below 19 trials registered for the treatment of BPD. The open-label, single-arm, single-centre, dose-escalation phase 1 trial (NCT01297205) for PNEUMOSTEM^®^, a human umbilical cord blood-derived MSC product, found delivery to be safe in extremely preterm infants [87]. Patients were monitored for 84 days following transplantation, and recorded adverse events were similar to an age-matched comparison group. There were nine patients enrolled in the trial, three of whom received the lower dose of 1 × 10^7^ cells per kg of body weight, and six of whom received the higher dose of 2 × 10^7^. No dose-limiting toxicities were observed. There were no statistically significant differences in disease severity scores in this study, confirming the need for a follow-up phase 2 study to determine efficacy [87]. A long-term follow-up study (NCT01632475) is currently being carried out on patients enrolled in this trial to confirm the safety of treatment long-term.

## 4. Delivery Methods

**Figure 1:**
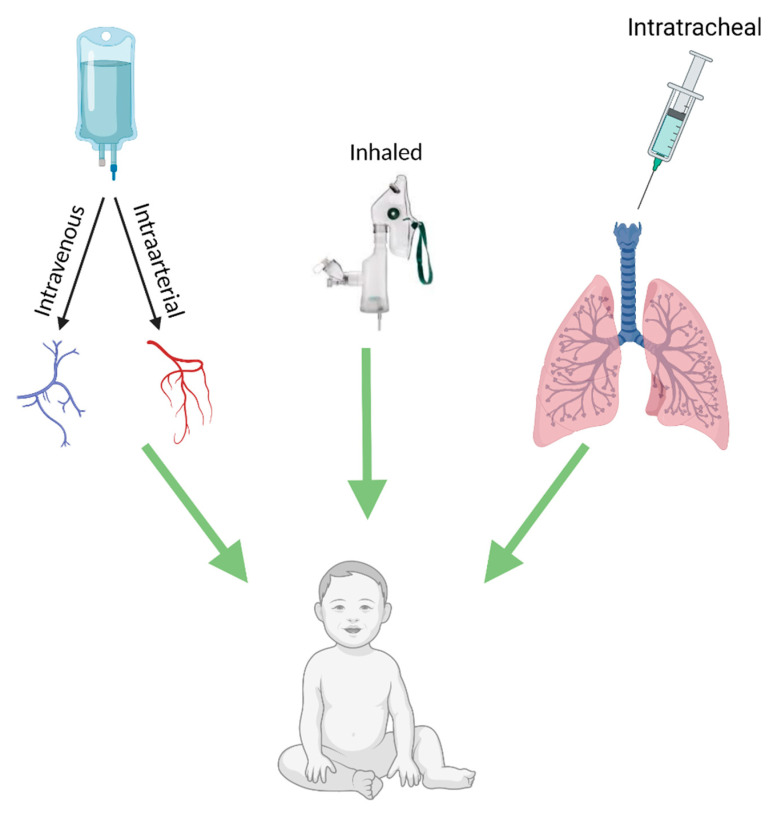
Potential routes of delivery for cell therapies to paediatric patients. Adapted from [23]. Created with BioRender.com (accessed on 21 July 2021).

While cellular therapy holds much promise for the treatment of respiratory disease, several hurdles need to be overcome to allow for the success seen in preclinical studies to translate to the clinic. These hurdles include the optimal dosage, cell type, cell source and route of administration appropriate for the disease type. An important aspect of treatment that has shown great variation in the literature is the optimal route of administration. Routes of administration for cellular therapies that have been employed in both preclinical and clinical studies include systemic delivery through the vascular route by intravenous (IV) or intra-arterial infusion or local delivery via intratracheal delivery (see Figure 1). [88]. The optimal route of administration for therapy must provide a uniform targeted delivery to the effected organ or tissue whilst preserving the integrity and quality of the cells in order to achieve optimal efficiency [23,89]. An important aspect for assessing the optimal delivery route is the biodistribution of the cells following delivery. Systemic delivery provides a broad distribution of cells throughout the body; however, entrapment of cells in the lungs has been found in a number of studies [90]. Other studies have suggested that this entrapment may be transient, with the majority of cells found to migrate to and remain in the spleen and liver [90]. Local delivery has been employed for a more targeted delivery to the affected organ or tissue and to overcome the potential side effects associated with systemic delivery [88].

Systemic delivery via the vascular route through intravenous or intraarterial infusion is a convenient, minimally invasive means of administration of a large number of cells, allowing for a greater biodistribution of cells directly into systemic circulation [88]. When cells are delivered via the intravenous route, the “first pass” effect occurs, during which cells may become entrapped in the lung [90]. While the majority of studies consider this a barrier to therapy, as cells do not reach the target organ, in the context of lung disease, this effect may, in fact, be desirable [91]. A study by Li and colleagues used near-infrared fluorescence imaging to track the biodistribution of MSCs labelled with NIR-DiR dyes in a rodent model of silicosis. They confirmed that rat BMSCs reached their peak in the lungs at 6 h post-transplant [92]. Although promising, the entrapment of cells in the pulmonary microvasculature due to their large diameter and expression of adhesion receptors may prevent cells reaching their targeted lung cell [93]. Another concern is that this entrapment in the vasculature may result in embolism formation. In a clinical trial administering MSCs for the treatment of Crohn’s disease, two participants were treated for venous thrombosis following IV infusion [23]. In a study by Lee and colleagues to determine potential efficacy of delivery of hMSCs to a rodent model of the infracted heart, they found that 80% of injected MSCs accumulated in the lung within minutes and embolized [94]. Furthermore, this entrapment in the lungs appears to be transient, with previous studies having shown that intravenously administered MSCs had relocated to the spleen and liver within 24 h of initial infusion [13]. The relative pharmacokinetics of systemic delivery means that pulmonary selectivity cannot be achieved, which, in turn, may present safety risks with potential for ectopic tumour formation [23].

There is a high cost associated with GMP manufacture of cellular therapies for use in the clinic. Local delivery offers potential for lower dosage requirements with equivalent or superior therapeutic efficacy for delivery of a larger dosage systemically [95]. Local delivery of MSCs includes delivery via direct injection including intramuscular or intraperitoneal or intratracheal, in the case of lung delivery, or via implantation of a scaffold that has cells embedded in it [88]. The majority of studies using cell-based therapy for respiratory disease have employed intratracheal delivery as the route of administration and have shown promising clinical benefits [27,33,44,87]. However, biodistribution studies of cell delivery to the lung have not been carried out. A study by Kim and colleagues was carried out in rodents to confirm real time migration and engraftment of MSCs delivered to the lung [93]. They found that delivery could be controlled by installation flow condition and the cell concentration for delivery and that targeted delivery to specific lung regions could be determined using their modelling system [93]. Although promising, a human model would need to be developed to determine requirements for targeted delivery. Furthermore, this confirms that effectiveness of installation may be compromised by the current methods, as modelling has not been carried out to confirm reliability. Intratracheal injection also poses a risk of delivery site morbidity caused by trauma.

### Future Prospects

The potential benefits to local delivery are undeniable; however, the procedure is typically highly invasive, requiring anaesthesia, and poses potential risks. Questions remain as to the distribution of cells on delivery. For advances to be made in developing sufficient drug delivery systems, a less invasive strategy with uniform targeted delivery needs to be employed. The application of a proven effective delivery method that has been used for centuries may be the answer for cell-based therapies. Inhaled therapies have been used for centuries [96] as a means of drug delivery for the treatment of lung disease. Inhalation of aerosols provides a rapid, high-pulmonary concentration of a therapy by direct delivery to the internal lumen of the airways and is associated with more rapid onset [95,97]. A recent study carried out by Halim and colleagues delivered aerosolised MSCs to an experimental rabbit model of allergen-induce asthma. They determined the effects of aerosolization of MSCs compared to pipette delivery to a culture flask; they found most of the cells were viable and observed similar morphology to the pipetted cells [98]. To determine the effects of MSCs on the asthma model, they delivered MSCs that were transfected with angiopoietin -1 and untreated MSCs by aerosolization to the animal. The levels of proinflammatory cytokines IL-4, TNF, TGF-β and MMP-9 were reduced following both treatments; however, angiopoietin-1-expressing MSCs showed a greater reduction of these proinflammatory cytokines [98]. This study confirmed the potential of aerosol delivery of cellular therapies as a feasible option, where cell quality and viability are maintained following delivery. The most common devices used for aerosol delivery to the lungs include pressurised metered dose inhalers (PMDIs), breath actuated PMDIs, soft mist inhalers and nebulisers [97]. PMDIs are commonly used for drug delivery of bronchodilators and corticosteroids to patients with asthma and COPD; however, they do not provide a feasible means of delivery for cellular therapies [95]. Nebulisers work through aerosolisation of a liquid solution or suspension to droplets and are suitable for delivery to patients receiving both invasive and noninvasive mechanical ventilation [99]. There are three main types of nebulisers: jet nebulisers, ultrasonic nebulisers and the newer vibrating mesh nebulisers (VMN) [100]. Ultrasonic nebulisers generate heat and have been associated with thermal inactivation of the drug for delivery and would not be suitable for cellular therapies [100]. Jet nebulisers require compressed gas to draw the drug from a reservoir through a baffle, which allows for smaller particles to be delivered, with the gas exiting towards the patient, whilst larger particles collide and are drawn back into the reservoir [101]. Jet nebulisers are reported to have low and varying levels of dose efficiency between brands and have a significant residual volume of the drug remaining in the device once dosing is complete (~40%) [102,103], due to the suspension buffer being easily aerosolised owing to its low viscosity; therefore, jet nebulizers are generally considered unsuitable for cells in suspension [104]. VMNs have been designed with an aperture plate with uniform-sized holes that vibrates to create a uniform dosage for delivery to the patient [101]. VMNs have minimal residual drug volume following use and have shown to have increased efficiency for drug delivery; however, no data yet exists demonstrating successful delivery of cells using VMN [102,105]. Nebuliser choice will be somewhat directed through the selection of patient intervention, with some nebuliser types either deemed unsuitable for use during certain interventions, e.g., JN being unsuitable for use during high flow nasal therapy, or delivery will be too low to economically or clinically rationalise its choice [104,106,107,108,109].

Delivery of aerosols to paediatric patients is complex, owing to the varying anatomical differences in the first few months of life through to childhood. Differences in airway sizes, lung volumes, respiratory rate and breathing patterns show vast variation in paediatric patients [110]. Furthermore, up to 18 months of age, children are obligate nose breathers, which is a further barrier to aerosol delivery [111]. Decreased dosage may directly correspond to these anatomical differences including reduced capillary size, lung surface area and body mass [102]. Device selection and patient interface are critical to improving efficacy, a face mask or transnasal delivery are the preferred route of administration for infants [102]. Réminiac and colleagues carried out a study using a primate model of an infant to determine the deposition of radiolabelled aerosols from different nebuliser types delivered via the nasal route [112]. In this study, 6-year-old female macaques were used as in vivo models of human infants; they received 2–8 L/min high gas flow with aerosol consisting of 150 mBq of 99m-technetium–diethylene–triamine–penta–acetic acid (Tc99m-DTPA) diluted in 0.9% sodium from either a jet or mesh nebuliser through a nasal cannula A Sophia Anatomical Infant Nose–Throat (SAINT) model connected to a respiratory pump was also used in this study as an in vitro model of a 9-month-old infant. Delivery using a face mask together with the mesh nebuliser and delivery using a face mask over nasal cannula with the jet nebuliser were used as controls. In this study, overall lung deposition was higher in the SAINT model compared to the macaque. At the same rate of 8 L/min, the lung deposition for each nebuliser within a nasal high-flow circuit was relatively low (0.46% jet nebuliser) (0.52% VMN). However, at lower flow rates of between 2 L and 4 L/min, the VMN achieved deposition of 3.3% and 4.2%, respectively, similar to the facemask control. The need for compressed air for the jet nebuliser means it cannot achieve these low flow rates, suggesting that the VMN is a more suitable option for intranasal delivery, capable of achieving similar rates seen for jet nebulisation with a face mask [112]. This confirms that nebuliser type and patient interface need to be carefully matched to achieve the greatest lung deposition. In a separate study, alveolar targeting in a paediatric model (Macaque) was confirmed through delivery of Modified Vaccinia Virus Ankara-mediated expression of green fluorescent protein in the alveolar space postaerosol administration [113]. In combination, these confirm the potential of intranasal delivery for an infant where delivery with a face mask is not tolerated well by the patient effecting the standard of care.

Regardless of the chosen route of administration, early identification of the one to be used in the development process is key for both reasons of continuity of technology throughout the cell therapy’s development and with an eye on future regulatory requirements as the therapy seeks approvals [114].

## 5. Conclusions

Cellular therapy presents a potential cure for currently incurable lung disease in both the adult and paediatric population. Current research has overlooked paediatric patients, focusing only on the adult form of the disease despite the majority of diseases occurring in children developing further into adulthood, resulting in long-term care. There is a clear need for research to move to the paediatric population. Current barriers to success of cellular therapies include the optimal dosage, cell type, cell source and route of administration. While the most current focus surrounds the cell product, the optimal route of delivery must be investigated to ensure integrity and efficiency of delivery whilst preventing unnecessary loss of the therapeutic product. Aerosol-based inhaled therapy presents a viable, noninvasive delivery method with more targeted delivery to the lung. Device selection and patient interface must be optimised to overcome the difficulties associated with delivery to paediatric patients.

## Figures and Tables

**Table 1:** Cellular based therapy for BPD.

NCT Number	Title	Study Results	Status	Condition	Therapy	Route of Administration	Study Design	Phase
NCT04255147	Cellular Therapy for Extreme Preterm Infants at Risk of Developing Bronchopulmonary Dysplasia	N/A	Not yet recruiting	Bronchopulmonary Dysplasia	Allogeneic Umbilical Cord Tissue-Derived MSCs	Single Dose Intravenous Infusion	Dose escalation study of ascending doses: 1 million cells 3 million cells 10 million cells (per kg of body weight)	1
NCT04062136	Umbilical Cord Mesenchymal Stem Cells Transplantation in the Treatment of Bronchopulmonary Dysplasia	N/A	Recruiting	Bronchopulmonary Dysplasia	Human umbilical cord-derived MSCs	Single Dose Intravenous Infusion	Safety and effectiveness study.	1
NCT04003857	Follow-up Study of Safety and Efficacy in Subjects Who Completed PNEUMOSTEM^®^ Phase II (MP-CR-012) Clinical Trial	N/A	Recruiting	Bronchopulmonary Dysplasia	PNEUMOSTEM^®^ Human umbilical cord blood-derived MSCs	Single Dose Intratracheal Injection	Follow-up of patients who completed Phase 2 trial to determine safety and efficacy.	2
NCT03873506	Follow-Up Study of Mesenchymal Stem Cells for Bronchopulmonary Dysplasia	N/A	Unknown	Bronchopulmonary Dysplasia	Human umbilical cord-derived MSCs	Single Dose Intravenous Infusion	Follow-up of patients who completed Phase 1 trial to determine safety and efficacy	1
NCT03857841	A Safety Study of IV Stem Cell-derived Extracellular Vesicles (UNEX-42) in Preterm Neonates at High Risk for BPD	N/A	Terminated	Bronchopulmonary Dysplasia	Extracellular vesicles from human bone marrow-derived MSCs	Single Dose Intravenous Infusion	Safety Study	1
NCT03774537	Human Mesenchymal Stem Cells For Infants At High Risk For Bronchopulmonary Dysplasia	N/A	Recruiting	Bronchopulmonary Dysplasia	Human Umbilical Cord-Derived MSCs	Intravenous Infusion	Dose escalation study of ascending doses: 1 million cells 5 million cells (per kg of body weight)	1
NCT02381366	Safety and Efficacy of PNEUMOSTEM^®^ in Premature Infants at High Risk for Bronchopulmonary Dysplasia (BPD)—a US Study	N/A	Completed	Bronchopulmonary Dysplasia	Human umbilical cord blood-derived MSCs	Not Listed	Dose escalation study 10 million cells 20 million cells (per kg of body weight)	1&2
NCT01897987	Follow-up Safety and Efficacy Evaluation on Subjects Who Completed PNEUMOSTEM^®^ Phase-II Clinical Trial	N/A	Completed	Bronchopulmonary Dysplasia	PNEUMOSTEM^®^ Human umbilical cord blood-derived MSCs	Single dose Intratracheal Injection	Long-term follow-up study to determine safety and efficacy of a Phase II trial (NCT01828957) until 60 months of corrected age.	N/A
NCT01828957	Efficacy and Safety Evaluation of Pneumostem^®^ Versus a Control Group for Treatment of BPD in Premature Infants	N/A	Completed	Bronchopulmonary Dysplasia	PNEUMOSTEM^®^ Human umbilical cord blood-derived MSCs	Single dose Intratracheal Injection	Safety and effectiveness study of a single dose.	2
NCT01632475	Follow-Up Study of Safety and Efficacy of Pneumostem^®^ in Premature Infants With Bronchopulmonary Dysplasia	N/A	Active Not recruiting	Bronchopulmonary Dysplasia	PNEUMOSTEM^®^ Human umbilical cord blood-derived MSCs	Single dose Intratracheal Injection	Long-Term Follow-Up Study of the Safety and Exploratory Efficacy of cohorts who received 1 × 10^7^ or 2 × 10^7^ (per kg of body weight)	N/A
NCT01297205	Safety and Efficacy Evaluation of PNEUMOSTEM^®^ Treatment in Premature Infants With Bronchopulmonary Dysplasia	Yes	Completed	Bronchopulmonary Dysplasia	Human Umbilical Cord Blood-Derived MSCs	Single dose Intratracheal Injection	Dose escalation study 10 million cells 20 million cells (per kg of body weight)	1
NCT01207869	Intratracheal Umbilical Cord-derived Mesenchymal Stem Cells for Severe Bronchopulmonary Dysplasia	N/A	Unknown	Severe Bronchopulmonary Dysplasia Extremely Premature Infants	Human Umbilical Cord-Derived MSCs	Instilled through a catheter into the endotracheal tube.	Single dose of 3 × 10^6^ cells (per kg of body weight)	1
NCT02381366	Safety and Efficacy of PNEUMOSTEM^®^ in Premature Infants at High Risk for Bronchopulmonary Dysplasia (BPD)—a US Study	N/A	Completed	Bronchopulmonary Dysplasia	Human umbilical cord blood-derived MSCs	Not Listed	Dose escalation study 10 million cells 20 million cells (per kg of body weight)	1&2
NCT01897987	Follow-up Safety and Efficacy Evaluation on Subjects Who Completed PNEUMOSTEM^®^ Phase-II Clinical Trial	N/A	Completed	Bronchopulmonary Dysplasia	PNEUMOSTEM^®^ Human umbilical cord blood-derived MSCs	Single dose Intratracheal Injection	Long-term follow-up study to determine safety and efficacy of a Phase II trial (NCT01828957) until 60 months of corrected age.	N/A
NCT01828957	Efficacy and Safety Evaluation of Pneumostem^®^ Versus a Control Group for Treatment of BPD in Premature Infants	N/A	Completed	Bronchopulmonary Dysplasia	PNEUMOSTEM^®^ Human umbilical cord blood-derived MSCs	Single dose Intratracheal Injection	Safety and effectiveness study of a single dose.	2
NCT01632475	Follow-Up Study of Safety and Efficacy of Pneumostem^®^ in Premature Infants With Bronchopulmonary Dysplasia	N/A	Active Not recruiting	Bronchopulmonary Dysplasia	PNEUMOSTEM^®^ Human umbilical cord blood-derived MSCs	Single dose Intratracheal Injection	Long-Term Follow-Up Study of the Safety and Exploratory Efficacy of cohorts who received 1 × 10^7^ or 2 × 10^7^ (per kg of body weight)	N/A
NCT01297205	Safety and Efficacy Evaluation of PNEUMOSTEM^®^ Treatment in Premature Infants With Bronchopulmonary Dysplasia	Yes	Completed	Bronchopulmonary Dysplasia	Human Umbilical Cord Blood-Derived MSCs	Single dose Intratracheal Injection	Dose escalation study 10 million cells 20 million cells (per kg of body weight)	1
NCT01207869	Intratracheal Umbilical Cord-derived Mesenchymal Stem Cells for Severe Bronchopulmonary Dysplasia	N/A	Unknown	Severe Bronchopulmonary Dysplasia Extremely Premature Infants	Human Umbilical Cord-Derived MSCs	Instilled through a catheter into the endotracheal tube.	Single dose of 3× 10^6^ cells (per kg of body weight)	1

## Data Availability

Not applicable.
